# Comprehensive evaluation of the effectiveness of endemic disease prevention and control in Jiangsu Province, China, 2013–2022

**DOI:** 10.3389/fpubh.2023.1271765

**Published:** 2023-11-03

**Authors:** Mao Liu, Yang Wang, Yunjie Ye, Yuting Xia, Li Shang, Zhen Ding, Peihua Wang

**Affiliations:** Environment and Health Institute (Endemic Disease Control Department), Jiangsu Provincial Center for Disease Control and Prevention, Nanjing, China

**Keywords:** endemic disease, comprehensive evaluation, prevention and control, weighted TOPSIS, weighted GRA, combined weights

## Abstract

As a strong economic and populous province in China, Jiangsu is home to four endemic diseases. Despite efforts in the past decade, the prevention and control of these four endemic diseases are not uniform because of the different etiological chains and influencing factors of these diseases. Among the evaluation methodologies for endemic disease control, only one method is currently available for each disease. In this study, we selected 14 indicators to comprehensively evaluate the effectiveness of endemic disease control in Jiangsu between 2013 and 2022. We improved the method for calculating the weights of the indicators and established a fuzzy comprehensive evaluation model based on the weighted Technique for Order Preference by Similarity to an Ideal Solution model and a weighted grey relational analysis model. The results of the comprehensive evaluation showed that the progress of endemic disease control in Jiangsu was not always in line with our expectations of improvement, with the top five years of better control occurring in 2015, 2013, 2021, 2022, and 2014. The results of the sensitivity analysis confirm the reliability and accuracy of these findings. We discovered that measures such as the reform of the salt industry, use of thyroid ultrasound, and new water supply projects for residents in Jiangsu affected the progress of endemic disease prevention and control. The tracking of endemic disease status should consider the potential effects of changes in policies implemented in other industries on endemic disease prevention and control. Additionally, the results of this study provide a theoretical basis for enhancing prevention and control practices in other regions of China.

## 1. Introduction

Endemic disease is defined as a disease that occurs in a population of a certain region due to natural or social factors without external input and exhibits local epidemiological characteristics (1). China is a large country with a wide range of endemic diseases (2). Jiangsu Province is located in eastern China and accounts for 1.06% of China’s total land area. Geomorphologically, Jiangsu comprises plains, hills, and mountains. Jiangsu ranked second in China in terms of gross domestic product (GDP) in 2022. At the end of 2022, Jiangsu had a population of 85.15 million permanent residents, ranking no. 4 in China, and was the most populous province in East China (3). Jiangsu is home to four endemic diseases: iodine deficiency disorders (IDD), endemic fluorosis, endemic arsenicosis, and water-source hyperiodinemic goiter (4–6). Jiangsu is home to 95 counties. IDD is prevalent in 93 counties (97.9%, 93/95). Endemic fluorosis is prevalent in 26 counties (27.4%, 26/95). Endemic arsenicosis is prevalent in 3 counties (3.2%, 3/95). Water-source hyperiodinemic goiter is prevalent in 2 counties (2.1%, 2/95).

The negative health effects of endemic diseases on residents have been well documented in several places worldwide. Iodine deficiency disorders can lead to fetal death and pediatric growth retardation (7) and may cause goiters in all populations (8). Excess iodine also poses health risks to the body, mainly to the thyroid, causing thyroiditis, hyperthyroidism, hypothyroidism, and goiter (9). Excess fluoride in drinking water harms the body, primarily in the teeth and bones and even the nervous and urinary systems in serious cases (10, 11). There is growing concern that children are sensitive to endemic fluorosis, especially regarding the effects of fluoride on their mental development (12). Excess arsenic in drinking water can cause serious chronic diseases such as keratosis pilaris and skin cancer (13). Considering its population size and GDP, endemic disease prevention and control are important in Jiangsu.

In the past decade, the central government has made significant investments in the implementation of endemic disease control programs. Jiangsu has successfully completed the 12th and 13th Five-Year Plans for the prevention and control of endemic diseases. In Jiangsu, a three-year action plan for the prevention and control of endemic diseases was implemented between 2018 and 2020. The National Health Commission issued the “Evaluation Methodology for Control and Elimination of Key Endemic Diseases (2019 Edition)” to evaluate the effectiveness of the control and prevention of each endemic disease (14). Every year, surveys are conducted to assess the status of the four endemic diseases in Jiangsu. From 2013 to 2022, we accomplished the arduous task of preventing and controlling endemic diseases in Jiangsu. According to the “Evaluation Methodology for Control and Elimination of Key Endemic Diseases,” each endemic disease in Jiangsu now meets the criteria for elimination or control. However, progress in the prevention and control of these four endemic diseases has not been uniform. Endemic disease control is not only a task for health departments; the effectiveness of this control is equally sensitive to policy changes and program implementation in other industries. In the last decade, there have been changes in the standard for the iodine level of salt, reform of the salt industry, changes in the way that drinking water is supplied to residents, and improvement in the health awareness of residents, among other changes, in Jiangsu (15–17). However, not all these changes have been favorable for endemic disease prevention and control. Due to changes in these potential factors, the progress of endemic disease control in Jiangsu does not always match our expectations for improvement, despite decades-long efforts to fight endemic diseases.

A suitable comprehensive evaluation method for many diseases has not yet been developed, and only one evaluation method is currently available for each disease among the evaluation methodologies for endemic disease control. The results of the control evaluation of a single disease cannot represent the overall status of endemic disease control in the province. To comprehensively evaluate the effectiveness of endemic disease control in Jiangsu between 2013 and 2022, we filtered 14 evaluation indicators for endemic disease control and established a fuzzy comprehensive evaluation model based on the weighted Technique for Order Preference by Similarity to an Ideal Solution (TOPSIS) and grey relational analysis (GRA) models. We improved the method of calculating the weights of the indicators using a combination of entropy and analytic hierarchy process (AHP) methods to calculate the combined weight, which allowed a more reasonable assignment of weights for each indicator. The results of the comprehensive evaluation model accurately demonstrated the dynamic changes in the effectiveness of endemic disease prevention and control over 10 years. Based on the evaluation results from this decade, we explored the social factors that may have affected the progress of endemic disease control in Jiangsu. Additionally, these findings provide recommendations for consolidating control achievements in Jiangsu and enhancing prevention and control practices in other parts of China.

The structure of this article is organized as follows. The second section introduces the data and methods, including the data collection and research methods. The research methods include the calculation of the combined weight values, calculation of the weighted fuzzy comprehensive evaluation model, cluster analysis of the comprehensive evaluation results, and sensitivity analysis of the comprehensive evaluation model. The third section describes in detail the results of the comprehensive evaluation model. The evaluation results are presented in the fourth section, while the fifth section details the conclusions and future plans for this study.

### 1.1. Literature review

Endemic disease is defined as a disease that occurs in a population of a certain region due to natural or social factors without external input and exhibits local epidemiological characteristics (1), which has have regional or endemic characteristics. Endemic diseases can be categorized into four pathopoietic categories, comprising geochemical diseases, specific lifestyle diseases, natural focus diseases, and endemic diseases of unknown etiology. Furthermore, China has some native genetic diseases, like β-thalassemia, which have never been incorporated in endemic disease management and control. Endemic diseases in China are defined as geochemical diseases, which differ from international definitions (18).

Endemic diseases are seriously prevalent in China, and the residents of 31 provinces suffer from them to varying degrees (19). Numerous endemic diseases are found in China, some of which are unique to the country, while others are also present in other countries. Nine types of diseases, including schistosomiasis, endemic fluorosis, Kashin-Beck disease, IDD, Keshan disease, endemic arsenicosis, water-source hyperiodinemic goiter, plague and burcellosis, were once included in the main management of endemic diseases for prevention and control. With the transition of administrative functions, schistosomiasis, plague, and brucellosis have been reclassified as communicable diseases. As a result, only the remaining six diseases are still under the management of endemic diseases for prevention and control.

Since 2000, Chinese Government has developed and released three Five-Year Plans consecutively to address the prevention and control of endemic diseases. Specifically, in 2018, the Central Committee of the Communist Party of China instructed on this matter. The State developed the “Three-Year Plan for Preventing and Controlling Endemic Diseases (2018–2020)” and coordinated special actions to combat them (20). The effectiveness of efforts to prevent and control endemic diseases has been evident (21).

The final evaluation results of the three-year campaign and endemic disease monitoring in 2021 showed that 2,799 IDD counties, 379 Kashin-Beck disease counties, 330 Keshan disease counties, 12 coal-fired pollution endemic arsenicosis counties, and 120 drinking water endemic arsenicosis counties or high arsenic areas have all met the elimination standard, with an elimination rate of 100%. The 171 coal-fired endemic fluorosis counties have all met the control or elimination standards, with a 100% rate for both [5]. Out of 1,042 drinking water endemic fluorosis counties, 1,008 have achieved the prevention and control measures standard, resulting in a 96.7% compliance rate (21, 22).

Geomorphologically, Jiangsu comprises plains, hills, and mountains (23). Jiangsu is home to four endemic diseases: IDD, drinking water endemic fluorosis, drinking water endemic arsenicosis, and water-source hyperiodinemic goiter (4–6). Jiangsu is home to 95 counties. IDD is prevalent in 93 counties (97.9%, 93/95). Endemic fluorosis is prevalent in 26 counties (27.4%, 26/95). Endemic arsenicosis is prevalent in 3 counties (3.2%, 3/95). Water-source hyperiodinemic goiter is prevalent in 2 counties (2.1%, 2/95). To date, the endemic disease trend in Jiangsu is under control.

#### 1.1.1. IDD

IDD is a syndrome that is induced by an inadequate intake of iodine due to the iodine deficiency of the external environment (5). The main cause of the condition is decreased thyroid hormone secretion. This leads to a range of mental development complications that negatively impact the quality of life of the population and represent a significant public health concern. IDD encompasses various diseases, such as endemic goiter, which is the most prevalent, endemic cretinism, miscarriage, neonatal congenital hypothyroidism, premature birth, deaf-mutism, congenital anomalies, stillbirth, subclinical cretinism, etc. IDD is the most prevalent and widespread disease in the world.

The results from the 2011–2021 monitoring demonstrate that IDD across the nation have achieved elimination levels. Furthermore, the goiter rate, referring to the rate of goiter among children aged 8 to 10 years, has been on a downward trend, decreasing from 2.4% in 2011 to 1.4% in 2021 and consistently remaining below 5%. There have been no new cases of endemic cretinism. The median urinary iodine level of children has declined from 238.6 μg/L in 2011 to 217.4 μg/L in 2021. Additionally, iodine nutrition was sufficient, meeting the criteria for determination based on a median urinary iodine level of 100–299 μg/L for children. The median urinary iodine level of pregnant women has decreased from 184.4 μg/L in 2011 to 172.0 μg/L in 2021, and iodine nutrition was in an adequate state (the criteria for determining the state of adequacy of iodine nutrition for pregnant women is a median urinary iodine level of 150 to 249 μg/L). The coverage rate of iodized salt and the consumption rate of qualified iodized salt have remained above 90%. However, the coverage rate of iodized salt has decreased from 98.0% in 2011 to 95.4% in 2021, whereas the consumption rate of qualified iodized salt has decreased from 95.3% in 2011 to 91.3% in 2021 (24).

#### 1.1.2. Endemic fluorosis

As a geochemical disease, endemic fluorosis that occurs in specific geographical environments. It arises from prolonged exposure to excessive quantities of fluorine in drinking water, food, and air, resulting in cumulative chronic toxicity throughout the body. Skeletal fluorosis and dental fluorosis are the primary clinical manifestations of endemic fluorosis. Fluorine naturally occurs in the form of a compound, not an element. Additionally, the majority of inorganic fluoride has the ability to dissolve in water and has a high boiling and melting point, resulting in fluorine’s extensive migration throughout air, water, soil, rocks, and animals, and it is easily obtained by humans. To prevent the occurrence of fluorosis, many countries have adopted the upper limit value of 1.5 mg/L for the fluoride content in drinking water recommended by the WHO (25). The national standard for fluoride concentration in drinking water is 1.0 mg/L in China and India. However, the limit is relaxed to 1.2 mg/L for small water supplies (< 1,000 m^3^ provided per day) in rural areas of China (26).

Monitoring findings in China showed that prevalence of dental fluorosis in children aged 8–12 years has decreased from 24.0% in 2012 to 10.8% in 2021, below the control level of 30%. The rates of water reform and rate of qualified water fluoride levels in drinking water endemic fluorosis counties have both increased significantly: the rate of water reform increased from 76.2% in 2012 to 99.0% in 2021, and the rate of qualified water fluoride levels increased from 74.9% in 2012 to 95.1% in 2021, with the highest rate of qualified water fluoride levels reaching 99.6% in 2020 (24).

#### 1.1.3. Endemic arsenicosis

Endemic arsenicosis is a chronic systemic poisoning caused by excessive arsenic intake resulting from prolonged exposure to high levels of arsenic in the natural environment (27). The primary pathogenesis involves hyperpigmentation or skin depigmentation, carcinogenesis, and palmoplantar keratoderma. Arsenic (As) is a prevalent element found in the environment, primarily in compound forms such as oxides, sulfides, hydrides of arsenic, and more. Four pathways expose humans to arsenic in the environment: iatrogenic intake, environmental pollution, occupational exposure, and life contact. Life contact is primarily responsible for the occurrence of endemic arsenicosis. This condition is mainly caused by consuming arsenic-contaminated groundwater and is common in all countries affected by arsenic poisoning (28). Moreover, in addition to arsenicosis caused by drinking water, there exists another form of endemic arsenicosis in remote mountainous regions affected by coal-fired pollution. This type of arsenicosis is known as coal-fired pollution endemic arsenicosis. Due to the burning of high-arsenic coal by residents, the food and air within their homes become polluted, resulting in arsenicosis.

Monitoring results in China showed that the detection rate of patients with arsenicosis has decreased significantly from 5.12% in 2012 to 0.48% in 2021, meeting the national elimination standard. In addition, from 85.7% in 2012 to 99.8% in 2021, the water conversion rate in drinking water-type arsenicosis areas has increased significantly. In addition, the rate of qualified water arsenic level has improved significantly from 81.3% in 2012 to 98.5% in 2021. There has been a significant increase in both the water conversion rate and the rate of qualified water arsenic level (24).

#### 1.1.4. Water-source hyperiodinemic goiter

Water-source hyperiodinemic goiter is caused by excessive intake of iodine, which results from the high iodine content in drinking water in the environment. China discovered water-source hyperiodinemic goiter for the first time. In recent years, high levels of iodine have been discovered in water sources located in several countries including Sri Lanka (29), Colombia (30), Germany (31), Sudan (32), and others (33). Regions with water-source hyperiodinemic goiter have been identified in 13 provinces of China. The mechanism by which high iodine intake leads to goiter formation is not yet fully understood. According to traditional pathogenesis, high iodine intake inhibits the synthesis or release of thyroid hormones by the thyroid gland, resulting in decreased hormone concentration in the blood. This, in turn, stimulates the pituitary gland to secrete thyroid stimulating hormone (TSH), which continuously stimulates the thyroid tissue, causing it to proliferate and enlarge, eventually forming a goiter.

Since 2012, China has conducted nationwide monitoring of areas with high iodine levels in water sources. The results indicated that the goiter rate in children aged 8 to 10 years peaked in 2014 at 9.3%, but has since decreased to less than 5% as of 2018. The median urinary iodine level in children decreased from 460.0 μg/L in 2012 to 287.2 μg/L in 2021, reaching the level of adequate iodine nutrition. Urinary iodine levels in pregnant women have been monitored since 2018, and the median urinary iodine has consistently remained at an appropriate level. From 2012 to 2017, the coverage rate of uniodized salt consistently exceeded 90%. However, in 2018, it sharply dropped to 46.2% before rebounding in the last three years. This drop can be attributed mainly to the abolition of the salt monopoly in the salt industry system reform. In addition, some areas with high levels of water iodization have yet to implement effective control measures (24).

The prevention and control of endemic diseases in China has always been centered on disease prevention and water diversion. The universal consumption of iodized salt is the primary measure for prevention and control of IDD. The prevention and control of high iodine goiter primarily requires consumption of uniodized salt and switching to a different water source. The drinking water endemic fluorosis and endemic arsenicosis is mainly to change the low fluoride and low arsenic water source, or to physicochemical treatment of fluoride (arsenic) water quality. Generally, these prevention and control measures are effective in achieving favorable outcomes in terms of disease prevention and control. However, some local areas face certain challenges that may affect the success of these measures. For IDD in China, two concerns are significant: the scope and concentration of universal salt iodization and the proper range of urinary iodine concentration for different populations. As the salt industry system reform progresses and salt varieties and markets become more accessible, there is a risk that the distribution of qualified iodized salt may decrease. This could lead to a decline in the median urinary iodine level in the population and even the resurgence of IDD (34). It is essential that sufficient attention is given to this issue. The prevention and treatment of Water-source hyperiodinemic goiter presents several challenges. Areas of high and low iodine levels are often intertwined, making it difficult to determine the appropriate extent of iodized salt supply interruption. Furthermore, changing the water in affected regions is challenging due to a lack of qualified water sources. Lastly, there exists no clear standard for when to resume the timely supply of iodized salt after a change in water supply. The main challenges associated with drinking water endemic fluorosis and endemic arsenicosis include low rates of water treatment in certain areas, low rates of regular operation, and low rates rate of qualified water after treatment. Much scientific research has been done on these endemic diseases, but many questions remain: First, there is a lack of appropriate physicochemical technology to remove fluoride, arsenic, and iodine from water for reuse in regions where it is difficult to find suitable water sources. Second, research is urgently needed to clarify the relationship between dietary iodine levels and thyroid disorders, including thyroid nodules and cancer, in order to promote the consumption of iodized salt. Third, the pathophysiology of fluorosis and arsenicosis is the most critical concern. Research on the pathogenesis of fluorosis and arsenicosis, especially on the mechanism of damage to non-specific target organs, is essential.

Although we have made some achievements in the prevention and control of endemic diseases after decades of efforts, the process of prevention and control of endemic diseases is affected by many factors mentioned above. How to further improve the prevention and control of endemic diseases and how to consolidate the prevention and control results already achieved has become a key issue for us.

## 2. Materials and methods

### 2.1. Data sources

All data were obtained from the White Paper on the Status of Endemic Disease Prevention and Control in Jiangsu Province from 2013 to 2022. Surveys for the four endemic diseases in Jiangsu are conducted according to the “National Monitoring Program for Iodine Deficiency Disorders” (35), “National Monitoring Program for Water-source High Iodine Areas” (36), “National Monitoring Program for Endemic Fluorosis” (37), and “National Monitoring Program for Endemic Arsenicosis” (38), respectively. Collectively, these programs use 15 monitoring indicators to assess the presence and severity of the endemic diseases. The National Health Commission issued the “Evaluation Methodology for Control and Elimination of Key Endemic Diseases (2019 Edition)” to assess the effectiveness of control and prevention of each endemic disease, with a total of 14 evaluation indicators (14). As per this methodology, we chose 14 out of the 15 monitoring indicators for evaluation. The evaluation indicators for iodine deficiency disorders (A1–A6, respectively) were the coverage rates of iodized salt and qualified iodized salt, the consumption rate of qualified iodized salt, the goiter rate of children aged 8–10 years, the urinary iodine level of children aged 8–10 years, and the urinary iodine level of pregnant women. The evaluation indicators of endemic fluorosis (B1–B2, respectively) were the rate of qualified water fluoride levels and the prevalence of dental fluorosis in children aged 8–12 years. The evaluation indicators for endemic arsenicosis (C1 and C2, respectively) were the rate of qualified water arsenic levels and the number of new cases of endemic arsenicosis. The indicators for evaluating water-source hyperiodinemic goiter (D1–D4, respectively) were the goiter rate in children aged 8–10 years, the coverage rate of non-iodized salt, the urinary iodine level in pregnant women, and the urinary iodine level in children aged 8–10 years.

### 2.2. Methods

#### 2.2.1. Weight determination

Previous studies have commonly used objective or subjective methods to determine indicator weights. However, subjectivity in the methods could result in biased weights, while relying solely on objectivity may lead to weights that are inconsistent with the actual importance of the indicators. To make the weight values more reasonable, we chose the classical entropy and AHP methods as the objective and subjective methods to calculate the comprehensive weight values, respectively.

The entropy and AHP methods were used to determine the weights of each evaluation index, denoted as $W_{i},V_{i}$ , respectively. The combined weights $Z_{i}$ are calculated using Equation (1), where *a* and *b* are the coefficients of the weights $a\in \left[0,1\right],b\in \left[0,1\right]$ . Generally, we consider *a = b =* 0.5 (39).


(1) $$Z_{i}=aW_{i}+bV_{i}$$

#### 2.2.2. Fuzzy comprehensive evaluation model

Multi-criteria decision analysis (MCDA) is primarily applied to solve complex problems with multiple objectives and multiple criteria. TOPSIS is a widely used classical multi-criteria decision-making method in many fields. However, the TOPSIS method still has shortcomings, such as failing to solve the problems of missing information and subjectivity. Studies have shown that the combined use of TOPSIS and other MCDA methods can be more effective in solving complex decision problems (40). To comprehensively evaluate the effectiveness of endemic disease control in Jiangsu Province from 2013 to 2022, we established a comprehensive evaluation model based on the weighted TOPSIS and GRA models.

The weighted TOPSIS and GRA models were used to calculate the evaluation values for each year, denoted as *C_i_* and *R_i_*, respectively, where greater *C_i_* and *R_i_* values indicated better effectiveness of endemic disease control. Equation (2) was used to calculate the evaluation value of the fuzzy comprehensive evaluation model, *M_i_*. We obtained the ranking for each year based on the *M_i_* value for each year, in descending order. A greater *M_i_* value indicated better endemic disease control. In Equation (2), *α* and *β* were the preference coefficients. Generally, *α = β =* 0.5 according to the fuzzy set theory (41, 42).


(2) $$M_{i}=\alpha C_{i}+\beta R_{i}$$

#### 2.2.3. Cluster analysis

Hierarchical clustering analysis was conducted to analyze the evaluation value *M_i_* of the fuzzy comprehensive evaluation model for each year. The Euclidean distance was used to measure the distance between clusters and the minimum distance was used to determine the type of distance between clusters.

#### 2.2.4. Sensitivity analysis

Generally, in Equation (2), *α = β =* 0.5. For *α + β =* 1, α and β could take any value between 0 and 1. To evaluate the reliability and stability of the research results, we designed nine (*α, β*) combinations of (0.1, 0.9), (0.2, 0.8), (0.3, 0.7), (0.4, 0.6), (0.5, 0.5), (0.6, 0.4), (0.7, 0.3), (0.8, 0.2), and (0.9, 0.1). The evaluation value *M_i_* of the fuzzy comprehensive evaluation model for different α and β combinations was calculated based on Equation (2), respectively. We then reordered *M_i_* and compared the rank changes in the control effectiveness.

## 3. Results

Table 1 lists the weight values of the entropy, AHP, and combined weight methods for the 14 evaluation indicators. The results of the entropy method showed that the indicators with the highest weights among the four endemic disease evaluation indicators were urinary iodine levels in pregnant women (A6), prevalence of dental fluorosis in children aged 8–12 years (B2), number of new cases of endemic arsenicosis (C2), and goiter rate in children aged 8–10 years (D1). The results of the AHP method showed that the indicators with the highest weight values among the four endemic disease evaluation indicators were the urinary iodine level of children aged 8–10 years (A4), rate of qualified water fluoride levels (B1), prevalence of dental fluorosis in children aged 8–12 years (B2), rate of qualified water arsenic level (C1), number of new cases of endemic arsenicosis (C2), goiter rate in children aged 8–10 years (D1), and the coverage rate of non-iodized salt (D2). The results of the combined weight method showed that the indicators with the highest weight values among the four endemic disease evaluation indicators were urinary iodine level in pregnant women (A6), prevalence of dental fluorosis in children aged 8–12 years (B2), number of new cases of endemic arsenicosis (C2), and goiter rate in children aged 8–10 years (D1).

**Table 1:** Weights of the evaluation indicators.

Names of diseases	Evaluation indicators	Code	*W_i_*	*V_i_*	*Z_i_*
Iodine deficiency disorders	Coverage rate of iodized salt	A1	0.0307	0.0170	0.0265
	Rate of qualified iodized salt	A2	0.0220	0.0170	0.0224
	Consumption rate of qualified iodized salt	A3	0.0315	0.0339	0.0379
	Goiter rate of children aged 8–10 years	A4	0.1081	0.1015	0.1213
	Urinary iodine level of children aged 8–10 years	A5	0.0349	0.0554	0.0509
	Urinary iodine level of pregnant women	A6	0.2818	0.0554	0.1447
Endemic fluorosis	Rate of qualified water fluoride levels	B1	0.0216	0.1015	0.0543
	Prevalence of dental fluorosis in children aged 8–12 years	B2	0.0368	0.1015	0.0708
Endemic arsenicosis	Rate of qualified water arsenic level	C1	0.0134	0.1015	0.0427
	Number of new cases of endemic arsenicosis	C2	0.2818	0.1015	0.1959
Water-source hyperiodinemic goiter	Goiter rate in children aged 8–10 years	D1	0.0602	0.1015	0.0905
	Coverage rate of uniodized salt	D2	0.0179	0.1015	0.0493
	Urinary iodine level in pregnant women	D3	0.0383	0.0554	0.0534
	Urinary iodine level in children aged 8–10 years	D4	0.0209	0.0554	0.0394

*W_i_*: weight values of the entropy method. *V_i_*: Weight values of AHP method. *Z_i_*: Combined weight values.

Table 2 shows in detail the evaluation values and ranks of the TOPSIS, GRA, and fuzzy comprehensive evaluation models on the effectiveness of endemic disease prevention and control in Jiangsu from 2013 to 2022. In the TOPSIS model, the top three years for prevention and control effectiveness were 2015, 2013, and 2021, respectively. The top three years in the GRA model were 2013, 2021, and 2015, while those in the fuzzy comprehensive evaluation model were 2015, 2013, and 2021, respectively.

**Table 2:** Evaluation of the effectiveness of endemic disease prevention and control in Jiangsu from 2013 to 2022.

Years	TOPSIS model		GRA model		Fuzzy comprehensive evaluation model	
	*C_i_*	Ranks	*R_i_*	Ranks	*M_i_*	Ranks
2013	0.4341	2	0.7710	1	0.6026	2
2014	0.2637	5	0.6610	5	0.4623	5
2015	0.6018	1	0.6990	3	0.6504	1
2016	0.1809	9	0.6510	7	0.4160	9
2017	0.1475	10	0.5950	10	0.3712	10
2018	0.2240	8	0.6230	8	0.4235	8
2019	0.2603	6	0.6230	8	0.4417	7
2020	0.2260	7	0.6580	6	0.4420	6
2021	0.3202	3	0.7250	2	0.5226	3
2022	0.2685	4	0.6940	4	0.4812	4

**Figure 1:**
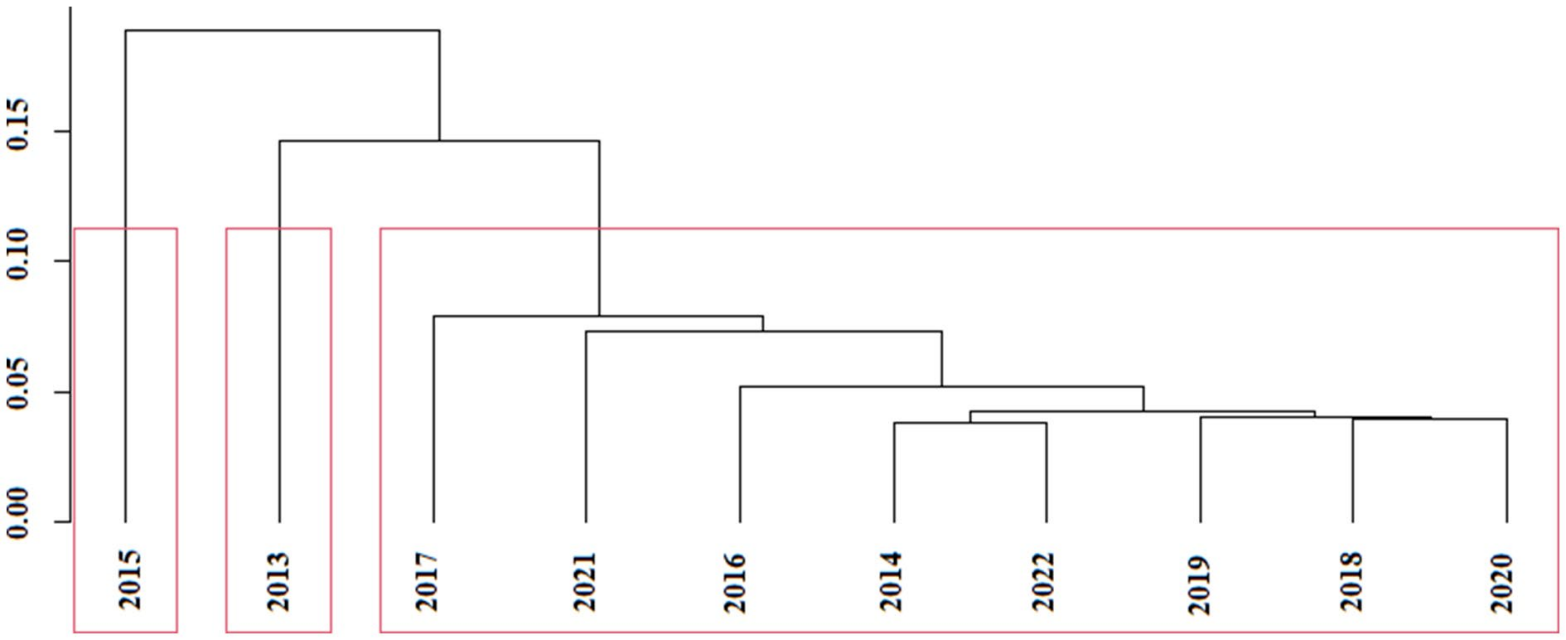
Results of hierarchical cluster analysis of the values of *M_i_*. Years within the same red box are combined into single categories.

Figure 1 shows the results of the cluster analysis of the evaluation values of the fuzzy comprehensive evaluation model. The effectiveness of endemic disease control from 2013 to 2022 was divided into three categories. Category I included 2015. Category II included 2013. Category III included 2017, 2021, 2016, 2014, 2022, 2019, 2018, and 2020. The effectiveness of endemic disease control in the three categories was in the following order: Category I > Category II > Category III.

**Figure 2:**
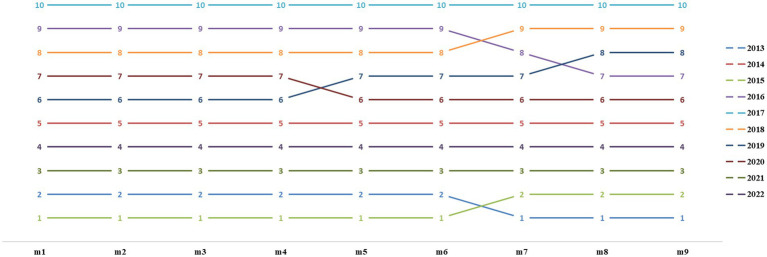
Comprehensive evaluation ranks for different values of *α* and *β* from 2013 to 2022. m1: *α* = 0.1 and *β* = 0.9; m2: *α* = 0.2 and *β* = 0.8; m3: *α* = 0.3 and *β* = 0.7; m4: *α* = 0.4 and *β* = 0.6; m5: *α* = 0.5 and *β* = 0.5; m6: *α* = 0.6 and *β* = 0.4; m7: *α* = 0.7 and *β* = 0.3; m8: *α* = 0.8 and *β* = 0.2; m9: *α* = 0.9 and *β* = 0.1.

Figure 2 demonstrates the changes in ranks in the fuzzy comprehensive evaluation model for nine values of *α* and *β*. When *β* changed from 0.4 to 0.3, the comprehensive evaluation values for 2013 and 2015 changed, with the rank of 2013 changing from no. 2 to no. 1 and the rank of 2015 changing from no. 1 to no. 2. The 2016 and 2018 rankings were exchanged. When *β* changed from 0.3 to 0.2, the ranks of 2019 and 2016 were exchanged. When *β* changed from 0.4 to 0.2, the rank in 2016 increased from no. 9 to no. 7. Overall, the ranks in the fuzzy comprehensive evaluation model did not change significantly due to changes in *α* and *β* values.

## 4. Discussion

This study constructed an evaluation system to assess the effectiveness of endemic disease prevention and control measures in Jiangsu, Eastern China, from 2013 to 2022. We improved the weight calculation method and calculated the weight of each index in the evaluation system using a combined weighting method. We proposed an improved comprehensive evaluation model based on the weighted TOPSIS and GRA models. The results of the comprehensive evaluation showed that the effectiveness of endemic disease control in Jiangsu did not improve as expected from 2013 to 2022. The years with the best control effectiveness were 2015, 2013, and 2021, while 2018, 2016, and 2017 showed the lowest effectiveness. The results of the sensitivity analysis confirmed the reliability and accuracy of the studying findings.

Endemic diseases in China are defined as geochemical diseases, which differ from international definitions (18). Because each endemic disease has different etiology and control measures, the effectiveness of endemic disease prevention and control in China is affected by a variety of factors. The National Health Commission of China has developed specific evaluation methods for each endemic disease based on its characteristics. We have not yet found another country in the world that has released a comprehensive evaluation method for the prevention and control of endemic diseases. Comprehensive evaluation of the effectiveness of multiple endemic disease control has multiobjective attribute, and it is usually difficult to balance between different objectives. A quantitative comprehensive evaluation method is necessary to improve the validity of the evaluation process and reduce subjectivity. Therefore, we developed a comprehensive evaluation model based on the weighted TOPSIS model and a weighted GRA model. Comprehensive evaluation methods based on MCDA have been applied in a number of fields (43–46), including evaluation of endemic disease control (47, 48). However, these evaluations have been conducted for one endemic disease rather than many, and there is a single evaluation model and weight determination methods. We have improved the method of calculating the weight values of the indicators, avoiding the possible shortcomings of using the entropy or AHP methods alone. Our study has improved the present methods of comprehensive evaluation, resulting in a more reasonable valuation outcome.

The entropy weight method objectively represents the practical status of endemic disease control over 10 years. We determined the weight of the AHP method based on the evaluation methodology. The weight values of the four indicators, A6, B1, B2, and C1, reflect the difference between the two weight calculation methods. The entropy and AHP methods underestimated and overestimated the weight values, respectively, which was consistent with the characteristics of the two methods. However, the combined weight method balanced the results of the entropy and AHP methods such that the weight values of the four indicators A6, B1, B2, and C1 were no longer overestimated or underestimated.

**Figure 3:**
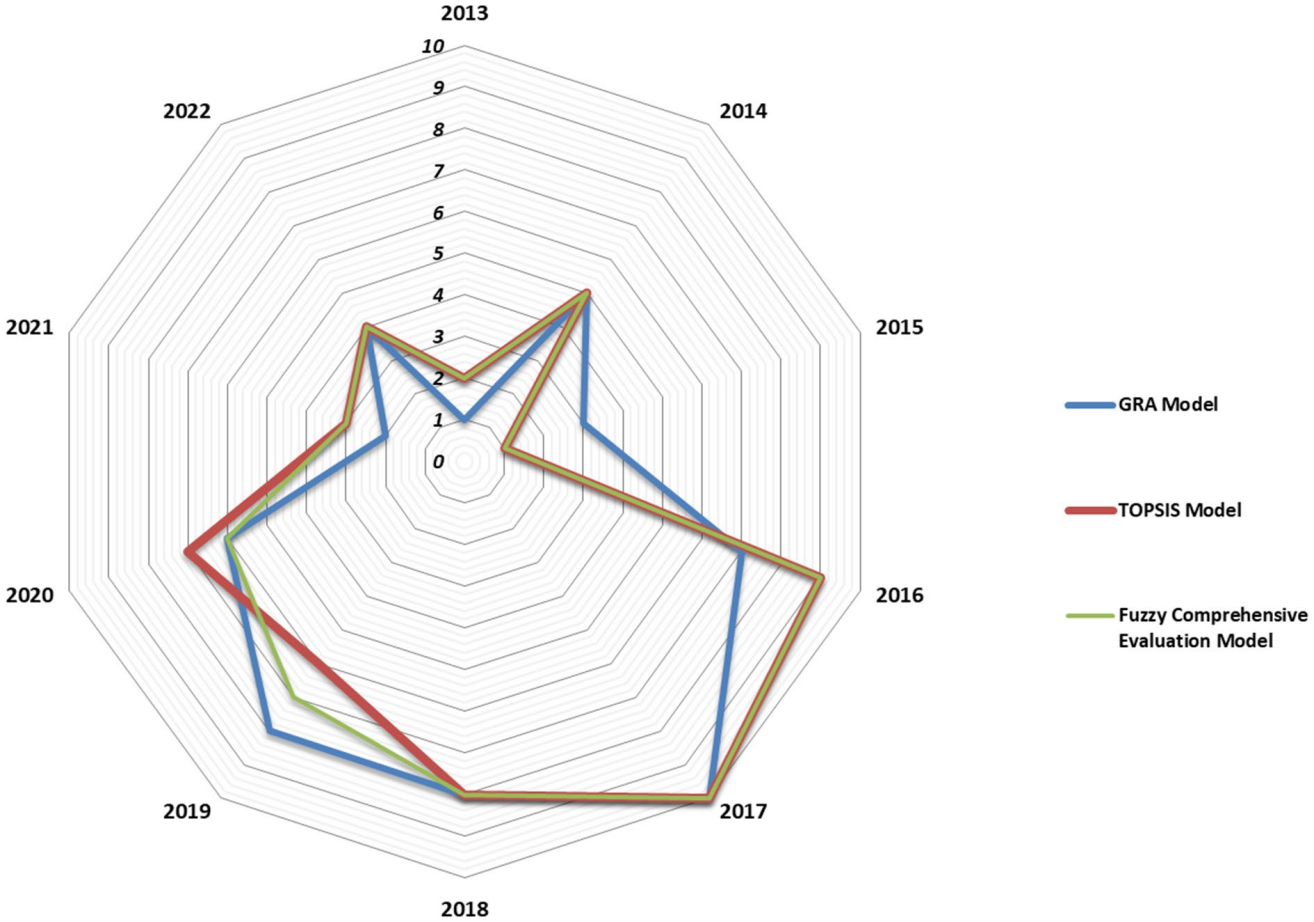
*C_i_*, *R_i_*, and *M_i_* rankings for each year from 2013 to 2022.

We calculated the evaluation value *M_i_* of the fuzzy comprehensive evaluation model based on the evaluation value *C_i_* of the weighted TOPSIS model and the evaluation value *R_i_* of the weighted GRA model. Figure 3 shows the rankings of *C_i_*, *R_i,_* and *M_i_* for each year from 2013 to 2022. The rankings from 2013 to 2022 did not show a linear trend despite continuous social and economic development, gradual improvement in endemic disease control measures, and a significant increase in funding for control during that decade. Instead, the top two years with better control were 2015 and 2013 and not 2022 and 2021. From 2015 to 2016, the rankings decreased rapidly from number 1 to number 9. In 2016, The State Council issued a plan to reform the Chinese salt industry (49). Salt companies from different regions can operate across regions, thus changing the previous model for salt market regulation. Salts from other regions have appeared in supermarkets and outlets in Jiangsu, including a large amount of non-iodized salt, which has affected the effectiveness of measures to prevent and control iodine deficiency disorders through universal salt iodization (USI) in IDD areas. The sum of the combined weight values of the salt-related evaluation indicators (A1, A2, A3, and D2) was 0.1361, demonstrating that changes in the four salt-related evaluation indicators could significantly affect the results of the comprehensive evaluation. However, the monitoring data showed that changes in salt-related evaluation indicators alone were insufficient to reduce the overall evaluation rank from 1 to 9.

Until 2016, palpation was the primary method used by endemic disease control programs in China to check goiter rates in children aged 8–10 years. Since 2016, the Center for Endemic Disease Control of the Chinese Center for Disease Control and Prevention has recommended the use of high-resolution B-ultrasound as a more precise method to measure thyroid volume to calculate the goiter rate in this population nationwide (50). The previous palpation method, which relied mainly on the experience and skill of endemic disease control professionals, produced unstable results and only visible goiters could be identified. In the monitoring data, A4 and D1 increased significantly in 2016 compared with 2015. The combined weight values of A4 and D1 were 0.1213 and 0.0905, respectively, which were the highest values. We argue that the nationwide reform of the salt industry and the use of high-resolution ultrasound machines have changed the effectiveness of endemic disease control. From 2017 to 2022, the comprehensive evaluation ranking showed a decreasing trend, which was consistent with our expectations. Beginning in 2017, the Water Resources Department has constructed new water-supply projects in the northern areas of Jiangsu. Endemic fluorosis, endemic arsenicosis, and water-source hyperiodinemic goiters all appear in the northern part of Jiangsu and are related to the quality of drinking water available to residents. New water supply projects have directly improved the quality of water used by residents in endemic areas, which directly reduced the harm caused by these three endemic diseases and indirectly improved the effectiveness of endemic disease prevention and control. The cluster analysis also divided the evaluation results from 2016 to 2022 into one category, consistent with the actual status of endemic disease prevention and control in Jiangsu during the same period.

## 5. Conclusion

The weighted fuzzy comprehensive evaluation model established in this study accurately demonstrated the dynamic changes in the effectiveness of endemic disease prevention and control in Jiangsu from 2013 to 2022. The top five years with the best endemic disease prevention and control were 2015, 2013, 2021, 2022, and 2014. Measures such as the reform of the salt industry, the use of thyroid ultrasound, and new water supply projects for residents during the past decade have changed the chain of endemic disease etiology and the progress of endemic disease control. However, the selected evaluation indicators did not include cost indicators such as the amounts of funds and personnel inputs, or social benefit indicators such as the response rate of residents’ knowledge of endemic disease prevention and control. Because of the difficulty in data collection, we did not include these indicators in the current study. Future tracking of the status of endemic disease control should collect as much information as possible on the evaluation indicators, including the prevalence of endemic disease control knowledge among residents, the amount of government financial input, and the level of education of endemic disease control professionals. It is necessary to consolidate the results of endemic disease control by considering the potential effects of policies changes in other industries on endemic disease control.

## Data availability statement

The raw data supporting the conclusions of this article will be made available by the authors, without undue reservation.

## Author contributions

ML: Formal analysis, Funding acquisition, Investigation, Methodology, Resources, Software, Writing – original draft. YW: Investigation, Methodology. YY: Investigation, Methodology. YX: Investigation, Methodology. LS: Investigation, Methodology. ZD: Writing – review & editing, Funding acquisition. PW: Writing – review & editing, Project administration.
